# Development of a Biomarker for Penconazole: A Human Oral Dosing Study and a Survey of UK Residents’ Exposure

**DOI:** 10.3390/toxics4020010

**Published:** 2016-05-13

**Authors:** Craig Sams, Kate Jones, Karen S. Galea, Laura MacCalman, John Cocker, Paul Teedon, John W. Cherrie, Martie van Tongeren

**Affiliations:** 1 Health and Safety Laboratory (HSL), Buxton SK17 9JZ, UK; craig.sams@hsl.gsi.gov.uk (C.S.); john.cocker@hsl.gsi.gov.uk (J.C.); 2 Centre for Human Exposure Science, Institute of Occupational Medicine (IOM), Edinburgh EH14 4AP, UK; karen.Galea@iom-world.org (K.S.G.); Laura.MacCalman@iom-world.org (L.M.); john.cherrie@iom-world.org (J.W.C.); Martie.VanTongeren@iom-world.org (M.T.); 3 School of Engineering and the Built Environment, Glasgow Caledonian University, Glasgow G4 0BA, UK; Paul.Teedon@gcu.ac.uk; 4 Institute of Biological Chemistry, Biophysics and Bioengineering, Heriot Watt University, Edinburgh EH14 4AS, UK

**Keywords:** penconazole, urine, biomarkers, fungicide, spray, residents, exposure, biological monitoring

## Abstract

Penconazole is a widely used fungicide in the UK; however, to date, there have been no peer-reviewed publications reporting human metabolism, excretion or biological monitoring data. The objectives of this study were to i) develop a robust analytical method, ii) determine biomarker levels in volunteers exposed to penconazole, and, finally, to iii) measure the metabolites in samples collected as part of a large investigation of rural residents’ exposure. An LC-MS/MS method was developed for penconazole and two oxidative metabolites. Three volunteers received a single oral dose of 0.03 mg/kg body weight and timed urine samples were collected and analysed. The volunteer study demonstrated that both penconazole-OH and penconazole-COOH are excreted in humans following an oral dose and are viable biomarkers. Excretion is rapid with a half-life of less than four hours. Mean recovery of the administered dose was 47% (range 33%–54%) in urine treated with glucuronidase to hydrolyse any conjugates. The results from the residents’ study showed that levels of penconazole-COOH in this population were low with >80% below the limit of detection. Future sampling strategies that include both end of exposure and next day urine samples, as well as contextual data about the route and time of exposure, are recommended.

## 1. Introduction

Penconazole (1-[2-(2,4-dichlorophenyl)pentyl]-1H-1,2,4-triazole) is a systemic triazole fungicide with preventive and curative properties for the control of powdery mildew [1]. It is used within the European Union on fruit and vegetable crops, particularly apples, hops and soft fruit; in the UK, in 2015, there were ten products authorised for professional use [2]. In the review of penconazole by the World Health Organisation in 1992, an acceptable daily intake (ADI) of 0.03 mg/kg body weight/day was determined [1], and a more recent re-evaluation by the European Food Safety Agency (EFSA) has proposed the same ADI value [3]. Potential human health effects arising from exposure have not been well characterised, although penconazole has been linked with an endocrine disrupting mode of action [4] and it is classified under the Global Harmonised System as H302: Harmful if swallowed (Acute toxicity, oral—Category 4) and H361: Suspected of damaging fertility or the unborn child (Reproductive toxicity—Category 2) [5]. In the UK, there remains public concern and debate regarding exposure to pesticides and the potential exposure of residents living close to agricultural land has been investigated recently [6,7,8]. A goal of this type of study is to determine appropriate biomarkers of pesticide exposure in human samples and to compare the relative amount of those biomarkers found to what might be anticipated from low-level human exposure at the ADI. Penconazole was identified in that study as one of the commonly used pesticides in UK orchards and, as such, there is potential that rural residents may be exposed. Penconazole is a widely used fungicide in the UK, with usage increasing since 2012, and it is currently the sixth most abundantly used pesticide in UK orchards (nearly 39,000 spray hectares in 2014 [9]).

**Figure 1:**
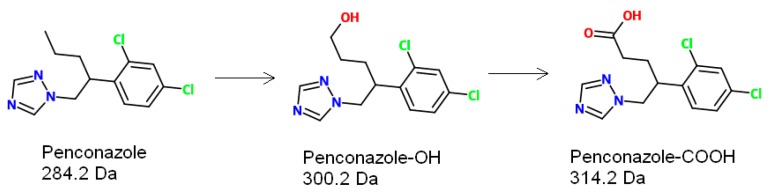
Chemical structure of penconazole and its metabolites.

Biological monitoring is a useful approach for determining exposure to chemicals and may be particularly useful for population-based exposure assessment where large numbers of non-invasive samples, such as urine, can be obtained relatively simply. It is also suitable for monitoring occupational exposure, since it enables the determination of the actual absorbed amount of chemical in an individual, accounting for any personal protective equipment such as protective clothing or respirator, dermal absorption or ingestion. However, such an approach requires an appreciation of metabolism and toxicokinetics (to identify a biomarker and an appropriate sample collection time), a suitable analytical method and an appropriate reference range in order to interpret the data. Animal studies have elucidated a number of potential urinary metabolites for penconazole [1] and an immunoassay has been reported for detecting a metabolite (4-(2,4-Dichlorophenyl)5-(H-1,2,4-triazol-1-yl)pentoic acid, penconazole-COOH) in spiked human urine [10]; however, no human exposure data have been published. From animal data, two oxidative metabolites were identified as candidate urinary biomarkers in humans (Figure 1). In addition, animal metabolism data indicate that excretion of penconazole and its metabolites was mostly complete within the first 24 h [1].

Human volunteer exposure studies can be helpful in establishing levels of biomarkers that may be expected following exposure to a defined dose of a substance. The Health and Safety Laboratory has previously conducted several such studies using a range of pesticides [11,12,13]. A similar approach has been used here, involving administering a single oral dose of penconazole to volunteers at the acceptable daily intake (ADI) of 0.03 mg/kg [1,3] and determining their urinary metabolite levels.

The objectives of the present study were to develop a robust analytical method for quantifying penconazole metabolites in human urine, then to determine biomarker levels in volunteers exposed to a single oral dose of penconazole at the ADI and, finally, to compare this data with measured metabolite levels in samples collected as part of a large investigation of rural residents’ exposure [6,7,8], in which penconazole was one of five target pesticides.

## 2. Experimental Section

### 2.1. Chemicals

Solid penconazole was obtained from QMX Laboratories (Thaxted, UK). Solid penconazole-COOH metabolite (4-(2,4-dichlorophenyl)5-(H-1,2,4-triazol-1-yl)pentoic acid) was obtained from Syngenta Crop Protection AG (Basel, Switzerland). A small aliquot of working solution containing penconazole-OH was obtained from Professor Silvia Fustinoni (University of Milan). All solvents and reagents used were of analytical grade.

### 2.2. Sample Preparation

Urine samples were stored frozen (−15 to −20 °C) and defrosted in a water bath prior to analysis. Calibration standards were prepared in glass screw-capped tubes using urine from an individual with no known overt exposure to penconazole by adding the appropriate volume of working solution (2 mg/L in methanol; stored refrigerated) to 2 mL urine to achieve a concentration range from 10—100 µg/L. Samples (2 mL) were analysed in duplicate and pairs of quality control (QC) samples, stored as frozen aliquots, were analysed after each block of five duplicate samples. One millilitre of an enzyme solution (250 µL β-glucuronidase from *Helix pomatia* (≥100,000 units/mL, Sigma Aldrich, Gillingham, UK) diluted in 50 mL 0.1 M acetate buffer, pH 5) was added to all samples, standards and QCs and incubated overnight at 37 °C.

To all tubes, 1 mL 0.2% phosphoric acid was added followed by liquid-liquid extraction using 4 mL ethyl acetate. Tubes were capped and mixed for 20 minutes, followed by centrifuging for 5 minutes. The organic layer (3 mL) was transferred to polypropylene tubes and evaporated to dryness under a stream of nitrogen. Samples were reconstituted in 100 µL mobile phase.

### 2.3. Sample Analysis

Samples were analysed using liquid-chromatography with tandem mass-spectrometry detection in selected reaction monitoring (SRM) mode (AB Sciex 3200 LC-MS/MS coupled to a Shimadzu Prominence LC). A 10-µL sample was injected onto a Genesis c18 column (100 × 2 mm, 3 µm; Jones Chromatography) and eluted using mobile phase containing 70:30 methanol: 10 mM ammonium formate (0.1% formic acid) at 0.2 mL per minute. The mass spectrometer was operated in positive electrospray ionisation mode and the SRM m/z transitions were 284.2/69.9 for penconazole, 300.1/70 for penconazole-OH and 314.0/70.1 for penconazole-COOH using 200 ms dwell times. Source temperature was set at 350 °C, ion spray voltage was 5000 V and gas flows were as follows: curtain gas 10 L/min; collision gas 5 L/min; ion source gas 1 and 2 20 L/min. Compound-specific mass spectrometer voltages were obtained using the instrument’s autotune function.

### 2.4. Method Characteristics

**Figure 2:**
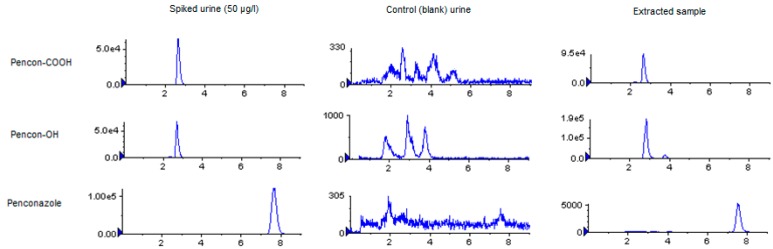
Chromatograms of a spiked urine sample (50 µg/L), a blank (control) urine sample and a volunteer sample (extracted), showing extracted traces for penconazole-COOH, penconazole-OH and penconazole.

The method was linear beyond 100 µg/L (least squares regression coefficient >0.99). Repeated analysis of QC samples gave inter-assay variations of 20% for penconazole and 15% for penconazole-COOH. Intra-assay variations were below 10%. Due to the very limited amount of penconazole-OH available, this compound was not added to QC material; however, calibration standards were run at the end of the batch to check that instrumental drift was acceptable. Detection limit for penconazole-COOH was determined as 0.25 µg/L based on three times signal to noise. Example chromatograms are given in Figure 2.

### 2.5. Creatinine Analysis

Creatinine was determined in all urine samples using an automated alkaline picrate method (Jaffe reaction) using a Pentra 400 clinical analyser (ABX, France) [14]. The coefficient of variation for within-day analysis was 1.5% and for between-day analysis was 3% at 6 mM. Data were adjusted for creatinine levels to account for hydration status and creatinine-corrected results were used for all data analysis.

### 2.6. Volunteer Study

The protocol used in this study was approved by the University of Sheffield Research Ethics Committee (study number HSL09, 2014). After giving informed written consent, three volunteers were given a single oral dose of penconazole at the ADI (0.03 mg/kg) dissolved in ethanol and diluted with a soft drink. Volunteer details are shown in Table 1. Total urine excreted was collected into a series of labelled containers for 24 h pre-dose and 48 hours post-dose. Pre- and post-dose collections covered the time periods 0–2, 2–4, 4–6, 6–8, 8–12, 12–20, 20–24 h with the post-dose collections additionally covering 24–28, 28–32, 32–36, 36–44 and 44–48 h post-exposure. The midpoint of each time period was used when data were plotted on graphs. The volume of each sample was recorded and an aliquot retained for analysis.

### 2.7. Residents’ Study

A study was conducted of residents living within 100 m of agricultural land that had been identified as likely to be subject to pesticide spraying. The overall study design has been described in detail elsewhere [7,8]. The study received full ethical approval by the NHS South East Scotland Research Ethics Committee (SESREC) 3 (study number 10/S1103/63). In brief, sample and data collection took place in three major arable crop growing and orchard areas in Great Britain: East Lothian, Kent, and Norfolk. Farmers were recruited into the study if they were likely to spray their agricultural crops with selected pesticides (captan, chlormequat, chlorpyrifos, cypermethrin and penconazole) and there were residential areas within 100 m of the fields being sprayed. The farmers provided details of their pesticide usage. Residents (adults aged 18 years and over and children in their care aged 4-12 years) were invited to participate in the study if they lived within 100 m of any field belonging to a enlisted farm. Participants provided informed written consent. First morning void urine samples were collected within two days after a spray event from participating residents, as well as a number of first morning void samples that were not associated with spray events (background samples collected during and outside of the spray season, with the spray season being taken to be March–August, 2011 and 2012). These urine samples were frozen soon after collection and stored at −15 to −20 °C prior to analysis. Urine samples associated with a spraying event were analysed only for the relevant pesticide(s) sprayed. Background samples were measured for all pesticides selected for the study. Participants were also asked to complete a questionnaire about the previous two days at the time of providing each urine sample; providing information on home and para-occupational pesticide usage, outdoor and indoor activities and their duration as well as home grown produce consumption.

The analytical methods and sample results for chlormequat, chlorpyrifos, captan and cypermethrin are described in detail in the project report [15]. The analytical method for penconazole (as detailed here) was also described there although samples were only analysed for penconazole-COOH and were not hydrolysed (the analysis took place before the volunteer study). Penconazole-COOH was quantified using multipoint matrix-matched calibration curves (including a blank) and QC samples (matrix spikes) were run every five samples (coefficient of variation = 14.9%, *n* = 308). Mean values were reported from duplicate analyses. Urine samples with detectable penconazole-COOH were reanalysed at intervals to assess sample stability. No sample degradation was observed over the evaluation period of 31 months.

## 3. Results

### 3.1. Volunteer Study

**Figure 3:**
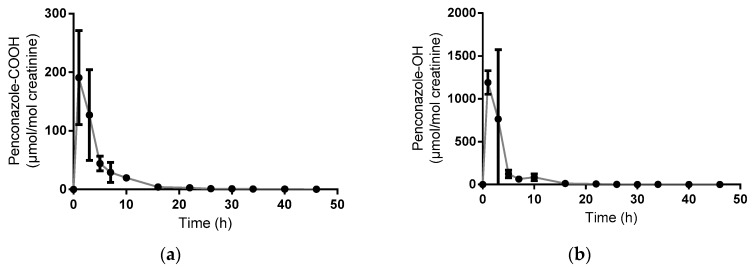
Urinary excretion of penconazole metabolites (penconazole-COOH (**a**) and penconazole-OH (**b**)) in hydrolysed urine (mean +/- standard deviation; *n* = 3). One volunteer did not produce a sample at the 2–4 hour time-point.

Only one of the pre-dose samples showed detectable penconazole-COOH levels (0.3 µmol/mol creatinine; 4.5% of samples collected), whereas 73% of pre-dose samples were detectable for hydrolysed penconazole-OH (up to 8.2 µmol/mol creatinine). Post-dose urinary excretion of both penconazole metabolites was complete within 48 hours of the oral dose. Quantifiable levels of metabolites (penconazole-OH and penconazole-COOH) were found in all but one of the volunteer exposure samples (see example in Figure 2), although levels were back within the pre-dose range in the latter sample collections. Figure 3 shows the time course of urinary excretion of both metabolites following hydrolysis of urine with β-glucuronidase. Peak urinary concentrations were found between 0–2 h post-dose.

**Figure 4:**
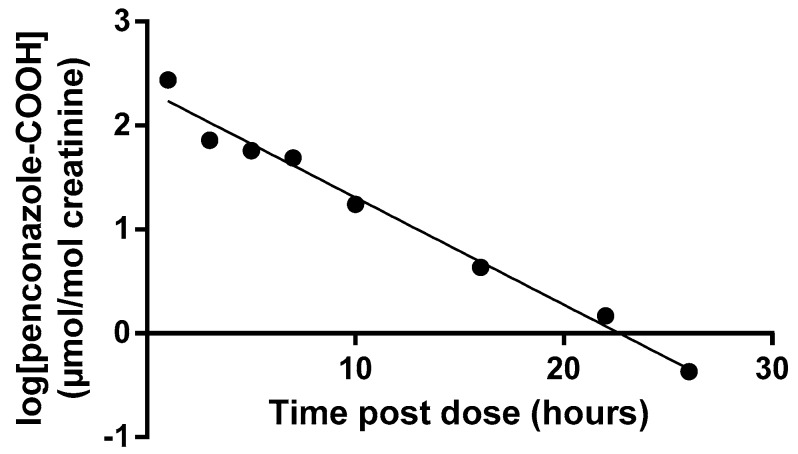
Half-life plot of excretion of penconazole-COOH. Data from volunteer C shown as an example (calculated half-life 2.9 h).

The mean peak penconazole-OH and penconazole-COOH concentrations were 1291 µmol/mol (1177 µg/L) and 214 µmol/mol (200 µg/L), respectively (hydrolysed). Excretion half-lives are shown in Table 2 with an example plot for a single volunteer shown in Figure 4.

Mean recovery of the administered dose was 47% (range 33%–54%) in urine treated with glucuronidase to hydrolyse any conjugates. Data for hydrolysed urine are shown in Table 3.

**Figure 5:**
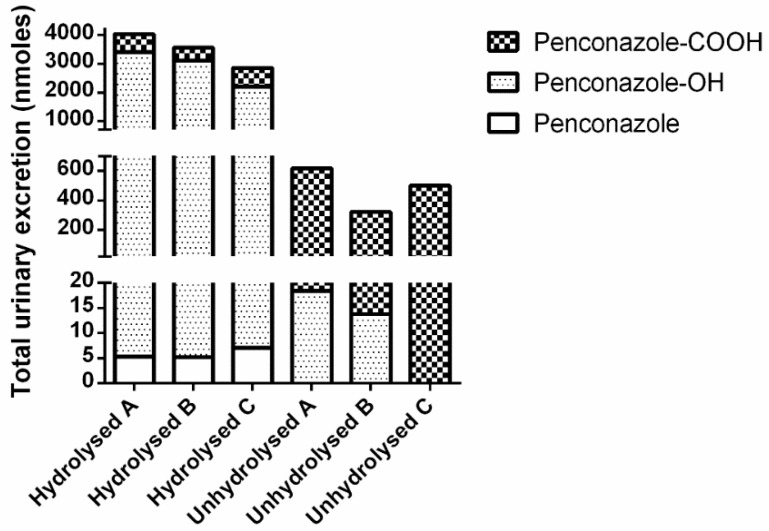
Comparison of recovered urinary metabolites (total nmoles in 48 hours post-dose) for both hydrolysed and unhydrolysed analysis (all volunteer data normalised to 70 kg body weight).

Penconazole-OH was the major metabolite in hydrolysed urine, comprising approximately 80% of the total metabolites. Very low levels (less than 0.5% of the dose) of un-metabolised penconazole were recovered. In contrast, un-hydrolysed urine contained very different ratios of metabolites (Figure 5). Less than 1% of the penconazole-OH metabolite was present in urine as un-conjugated metabolite. One volunteer (C) did not excrete any detectable un-conjugated penconazole-OH. However, 80% (range 69%–97%) of penconazole-COOH was present in urine as free metabolite.

Due to the novel biomarkers monitored and the lack of commercial standards or quality assurance samples, a subset of volunteer study samples were also analysed by Professor Silvia Fustinoni (University of Milan). There was reasonable agreement between the two laboratories for penconazole-OH (slope = 0.885, r = 0.897, *n* = 14) and penconazole-COOH (slope = 1.244, r = 0.872, *n* = 17).

### 3.2. Residents’ Study

Penconazole was sprayed only in the orchards recruited in the Kent region of this particular study. Nine farms participated and 33 spray events involving this pesticide were noted during the two spray seasons monitored. Forty-eight adults and six children were eligible participants providing penconazole spray event related urine samples.

Before any data analysis of the urinary metabolite concentrations took place, a number of necessary exclusions were made based on pre-defined eligibility for inclusion in the study (at least one spray event related sample and at least one background). Urine samples where the creatinine level was below 2 or greater than 30 mmol/L (0.23 g/L and 3.39 g/L, respectively) were excluded [7,8]. From the penconazole spray events, 89 urine samples were collected from residents. In addition, 556 within season and 483 outwith season background urine samples were collected from the wider study population.

A very high proportion of samples was below the analytical limit of detection (LOD) for penconazole-COOH (81% following spray events and 89% at other times) and so only the proportion of detects, the 95^th^ percentile and the maximum level are reported in Table 4. Due to the high proportion of non-detects no further analysis of questionnaire responses or modelling was performed [6].

## 4. Discussion

Penconazole is a widely used fungicide in the UK [9] and this paper presents the first journal-published human data demonstrating the urinary excretion of two metabolites of penconazole that represent a significant percentage of an orally administered dose (penconazole-OH and penconazole-COOH). Thus, these metabolites could be utilised as markers of systemic exposure to penconazole. Penconazole-OH was excreted almost entirely as conjugates, whereas penconazole-COOH was excreted primarily (>69%) as the free form in human volunteers. Fustinoni has presented work at the 31st ICOH conference [16] and the 2015 International Congress on Rural Health [17] looking at exposures to agriculture workers. The peak excretion values measured by Fustinoni were 258 μg/L and 20 μg/L for penconazole-OH and penconazole-COOH, respectively compared with 1291 μg/L and 232 μg/L, respectively, following a single oral dose at the ADI (0.03 mg/kg/day), all after hydrolysis. Based on these comparisons, and the demonstration in our work that hydrolysed penconazole-OH and penconazole-COOH are major metabolites in human urine, the results of Fustinoni indicate that the internal dose to workers under the conditions of their study was likely to be less than 0.03 mg/kg/day.

The analytical method presented here for penconazole-COOH (the lack of a standard prevented characterisation of the method for penconazole-OH) is sensitive (LOD 0.25 µg/L, at least 300 times lower than the peak urinary concentration following an ADI oral dose), reproducible (inter-assay variation < 15%, *n* = 308) and robust (over 1100 samples analysed over 31 months, no deterioration of samples upon frozen storage [15]). The LOD was comparable with that reported for other triazole fungicides [18] and the other pesticides measured in the residents’ study [7].

The volunteer study has demonstrated that both penconazole-OH and penconazole-COOH are excreted by humans following an oral dose, confirming the data presented by Fustinoni *et al.* [16,17]. Peconazole-OH is excreted largely conjugated and as such is a significant metabolite (25%–47% of the administered dose). Penconazole-COOH is excreted largely unconjugated and accounts for 7%–8% of the dose (with seemingly less variation between volunteers although the sample size is small). The ratio of the metabolites is similar to that found by Fustinoni [18] in vineyard workers exposed to tebuconazole, with a ratio of tebuconazole-OH : tebuconazole-COOH of 3.5:1 (after hydrolysis). We found the peak excretion of metabolites after an oral dose of penconazole occurred within 2 h and was followed by elimination with a half-life of <4 h, whereas studies of occupational exposure to tebuconazole found peak excretion occurred 16–24 h after exposure, possibly due to dermal exposure [18,19] where absorption, and hence excretion, can be significantly delayed. The short half-life of the penconazole metabolites after oral dosing would suggest a monitoring strategy for inhalation and oral exposures would be to collect end of exposure samples. Where there is potential for dermal exposure samples should also be collected the following day (e.g., first morning void), to determine if peak levels occur at a later interval (as seen with tebuconazole worker studies). However, the LOD for penconazole-COOH is almost three orders of magnitude lower than the peak level seen in the volunteer study so if residents were exposed to penconazole, even at levels below the ADI, metabolites should be detectable in urine samples collected within 4 to 5 half-lives; the volunteer study showed levels at least 10× the LOD at 24 h post-dose. Furthermore, it is likely that if residents were exposed to penconazole it would be mostly through dermal exposure [20] rather than ingestion, with the delay in peak exposure as seen in the tebuconazole study [18,19]. In these scenarios, collecting urine samples the following day would increase the likelihood of detection in samples collected the following day. However, the risk assessment model [6] suggested that the primary exposure route would be “inhalation following volatilisation of the pesticide after spray event” so both scenarios should be considered in designing a sampling approach in future studies.

The results from the residents’ study showed that levels of penconazole-COOH in this population were low with >80% below the LOD. This is supported by the pre-dose volunteer samples where there were 95% of results below the LOD. The maximum result from the residents’ study was 1.8 µmol/mol creatinine, which is at least 100× lower than the peak excretion after an oral dose at the ADI and is also less than the 12–20 h samples from volunteers (range 1.9–4.0 µmol/mol creatinine), which might better reflect the likely potential exposure of residents to any spray. The detection of low levels of unhydrolysed penconazole-COOH in volunteer samples up to 48 h after dosing indicates that general population levels are genuinely low and not due to an inability of the methodology to detect exposure. The penconazole results and comparisons with levels predicted by regulatory models are discussed in more detail elsewhere [6].

## 5. Conclusions

The work presented here demonstrates that both penconazole-COOH and penconazole-OH are specific human metabolites of penconazole. Following hydrolysis, penconazole-OH is the major metabolite but may be more variable between individuals compared to penconazole-COOH. The lack of availability of a penconazole-OH standard prevented its analysis in the residents study but it is clear that, as a major metabolite, it should be included (with hydrolysis) alongside penconazole-COOH in any future biomonitoring studies of penconazole. The volunteer study has demonstrated that an oral dose is readily excreted with a half-life of less than four hours. Although this half-life may be relevant for looking at dietary exposures, the toxicokinetics for dermal exposures are likely to be significantly delayed (due to delayed absorption). A sampling strategy that includes both end of exposure and next day urine samples, as well as contextual data about the most likely route of exposure (depending on the nature of the task or incident) and the time of the exposure relative to the samples taken is recommended.

## Figures and Tables

**Table 1:** Details of the volunteers for the oral dose study.

Code	Sex	Height (m)	Weight (kg)	Age (Years)	BMI*
A	M	1.78	72	59	22.7
B	F	1.58	62	50	24.8
C	M	1.88	83	62	23.5

* Body Mass Index.

**Table 2:** Urinary metabolite excretion half-lives calculated from three volunteers exposed to a single oral dose of penconazole at the acceptable daily intake (ADI) of 0.03 mg/kg body weight.

	Mean Half-Life (h)	Range (h)
Penconazole-OH	3.1	2.6–3.5
Penconazole-COOH	3.7	2.9–4.0

**Table 3:** Recovery of urinary metabolites within 48 h of an oral dose, following hydrolysis.

Code	% Administered Dose Recovered in Urine	Metabolite Fraction of Total		
		Pencon-OH	Pencon-COOH	Penconazole
A	53	0.85	0.15	0.001
B	54	0.87	0.13	0.001
C	33	0.77	0.23	0.002

**Table 4:** Summary of the urine results (unhydrolysed penconazole-COOH, µmol/mol creatinine) from the residents’ study [15].

Sample Type	N	N < LOD	%<LOD	Maximum	95th Percentile *
Background sample—Outwith spray season	483	427	88	1.73	0.22
Background sample—Within spray season	556	500	90	1.19	0.29
After spray event	89	72	81	1.84	0.32

N = number; LOD = Limit of Detection; * of all data.
